# Freestanding midwifery units versus obstetric units: does the effect of place of birth differ with level of social disadvantage?

**DOI:** 10.1186/1471-2458-12-478

**Published:** 2012-06-22

**Authors:** Charlotte Overgaard, Morten Fenger-Grøn, Jane Sandall

**Affiliations:** 1 Department of Health Science and Technology, Aalborg University, 9220Aalborg, Denmark; 2 Research Unit for General Practice, Aarhus University, Denmark; 3 King’s College,Women’s Health Academic Centre King's Health Partners, London, UK

**Keywords:** Childbirth, Freestanding midwifery unit, Social inequity, Birth outcomes, Social position, Level of education, Low risk women

## Abstract

**Background:**

Social inequity in perinatal and maternal health is a well-documented health problem even in countries with a high level of social equality. We aimed to study whether the effect of birthplace on perinatal and maternal morbidity, birth interventions and use of pain relief among low risk women intending to give birth in two freestanding midwifery units (FMU) versus two obstetric units in Denmark differed by level of social disadvantage.

**Methods:**

The study was designed as a cohort study with a matched control group. It included 839 low-risk women intending to give birth in an FMU, who were prospectively and individually matched on nine selected obstetric/socio-economic factors to 839 low-risk women intending OU birth. Educational level was chosen as a proxy for social position. Analysis was by intention-to-treat.

**Results:**

Women intending to give birth in an FMU had a significantly higher likelihood of uncomplicated, spontaneous birth with good outcomes for mother and infant compared to women intending to give birth in an OU. The likelihood of intact perineum, use of upright position for birth and water birth was also higher. No difference was found in perinatal morbidity or third/fourth degree tears, while birth interventions including caesarean section and epidural analgesia were significantly less frequent among women intending to give birth in an FMU. In our sample of healthy low-risk women with spontaneous onset of labour at term after an uncomplicated pregnancy, the positive results of intending to give birth in an FMU as compared to an OU were found to hold for both women with post-secondary education and the potentially vulnerable group of FMU women without post-secondary education. In all cases, women without post-secondary education intending to give birth in an FMU had comparable and, in some respects, more favourable outcomes when compared to women with the same level of education intending to give birth in an OU. In this sample of low-risk women, we found that the effect of intended place on birth outcomes did not differ with women’s level of education.

**Conclusion:**

FMU care appears to offer important benefits for birthing women with no additional risk to the infant. Both for women with and without post-secondary education, intending to give birth in an FMU significantly increased the likelihood of a spontaneous, uncomplicated birth with good outcomes for mother and infant compared to women intending to give birth in an OU. All women should be provided with adequate information about different care models and supported in making an informed decision about the place of birth.

## Background

Social inequity in perinatal and maternal health is a well-documented health problem [1] affecting women the world over. Systematic disparities in health associated with social determinants [2] are still seen in societies with high levels of social equality. Even in the Nordic countries with their comprehensive public health care and welfare systems, social factors exert a strong influence on both maternal and perinatal birth outcomes [1,3].

Socially disadvantaged women, as defined by factors such as low levels of education, employment, income, or residence in a deprived area, suffer increased morbidity and mortality during childbirth [1,4] when compared to women from socially advantaged backgrounds. Their infants have higher perinatal and neonatal morbidity and mortality [3-9] and are more often born preterm [10-13], with lower Apgar scores and birth weight [4,9,14-16] and are overrepresented [17,18] in neonatal units.

The incidence of epidural analgesia [19,20], use of an upright birth position [21], caesarean section and other birth interventions have also been suggested as being affected by social inequality, but results on caesarean section are conflicting with some studies finding a higher [22,23] and others a lower likelihood among disadvantaged women [24-29]. It is unclear whether this inconsistency in findings for caesarean section and epidural is due to differences in the organisation of maternity care services (private/public) [25,28-30], hospital specialisation level [31], and the type of lead caregiver (obstetrician/midwife) [32]. It may be noted, though, that the use of birth interventions is more widespread in societies with high levels of hospitalisation and specialisation and where private health services are prevalent [25,28-31].

It has been argued that disadvantaged pregnant women perceive themselves as having little knowledge and little choice, and that they have considerable faith in medical “experts” [33], and are more positive towards interventions and use of medical pain relief compared to advantaged women [34]. In this perspective, disparities in the use of intervention, pain relief and birth position are seen to reflect different preferences between the two groups of women. However, Green et al. [35,36] have contested this perception while Lazarus has argued that insufficient attention is given to how social restraints and conditions impact on women’s expectations and experiences [37]. We find it likely, as argued by de Jorge [21], that some care options are offered less frequently to disadvantaged women while health professionals tend to offer more positive responses to the wishes and demands of advantaged, confident and articulate women [28,38]. They may also generally receive a higher level of continuity of care [39], higher quality care and be prioritised over disadvantaged women [40].

The complex relationship between social disadvantage and birth outcomes is confounded by the influence of several factors such as stressful life conditions, life style, health behaviours and their accompanying/underlying medical conditions [41]. Despite an overall increased risk of complications, the majority of disadvantaged women enter spontaneous labour at term without having developed maternal or perinatal complications and are thus categorised as being at low risk of intrapartum complications. As population-based studies generally are not able to take into account differences in women’s obstetric risk factors [1,3-10,13,14,16-18], it is unclear whether social inequality persists among these women.

Obstetric units (OU) have today become the primary setting for birth in most middle- and high-income countries, often with all frontline care being provided by midwives. However, alternative birth settings such as freestanding midwifery units (FMUs) are also offered in several countries, including New Zealand [42], the United Kingdom [43], Canada [44], the United States [45], Italy [46], Germany [47], the Republic of South Africa [48], Brazil [49], Norway [50], in some of which childbirth policies aim to provide women with a choice of birthplace [51,52].

Generally, FMUs are based on a woman/family-centred philosophy and aim to provide supportive, individualised care and encourage spontaneous, vaginal birth [53]. They provide low-risk women with a choice among different models of intrapartum care. In sparsely populated areas, FMUs offer care closer to home (to low-risk women) [50], while in low-income countries they may provide women with affordable and accessible care [54,55].

The primary professional responsibility for care in FMUs is in the hands of midwives. All need for obstetrical, neonatal, and anaesthetic care requires ambulance transfer of the women and /or infant to an OU [56]. As acute perinatal and maternal complications may arise in spite of careful risk assessment of women, safety of FMU care has been a concern and until recently limited evidence has been available [57].

In 2011 the Birthplace in England Research Programme, an extremely large, prospective cohort study, found no significant differences in perinatal outcome between women intending to give birth in a FMU and women intending to give birth in an OU while the use of medical interventions and medical pain relief were significantly reduced among women receiving care from FMUs [58]. In our own recent study of FMU versus OU care in Denmark, we compared perinatal outcomes for low-risk women intending to give birth in an FMU and low-risk women intending to give birth in an OU. We also found no difference for perinatal outcomes while women in the FMU group had reduced maternal morbidity and fewer birth interventions [59].

Several studies document that the women rate their experience of care in terms of psycho-social outcomes more positively in midwifery units compared to OUs [43,44,60-63]. In our study of FMU care, we also found that the effect of FMU care on women’s birth experiences differed by women’s level of social disadvantage and that FMU care had a mitigating effect on the effect of social disadvantage on birth experience [63]. With this increased evidence on the safety and quality of care in midwifery units [64], it seems likely that more low-risk women will choose FMU settings for birth if they are available.

In general, non-OU settings for birth have been found to be the choice of the group of more mature, better-educated, middle-class women of socially privileged backgrounds [44,58,65-67]. However, proximity is also seen to exert a strong influence on women’s choice of birthplace [68-70]. With increasing distance between maternity units as a result of centralisation, the social characteristics of women choosing a non-OU service may become more mixed [68].

There is limited evidence concerning birth outcomes of FMU versus OU care for disadvantaged women. A systematic literature search identified only one study on perinatal and maternal outcomes in FMUs, which explored the interaction between birthplace and perinatal and maternal birth outcome. This study concluded that outcomes did not differ by women’s level of social disadvantages [71]. Our study of two FMUs located in community hospitals in peripheral, low education and low income areas, provides a rare opportunity to investigate the outcomes and suitability of FMU care for socially disadvantaged women.

### Objectives

The aim was to study the whether the effect of intended birthplace on perinatal and maternal morbidity, birth interventions and use of pain relief and upright position for birth among low risk women intending to give birth in two FMU versus two OU in Denmark differed by level of social disadvantage.

The study is reported in accordance with the STROBE requirements for observational studies [72,73].

### Study hypotheses

Our study of the literature led us to hypothesise that in the present sample of low risk women where all frontline care in both groups are provided by midwives in the context of a public health system, the effect of birthplace on perinatal and maternal morbidity would not differ by women’s level of education.

For disadvantaged women we hypothesised that FMU care, with its focus on social support, individualised care and shared decision-making, would support the likelihood of spontaneous, uncomplicated birth, water birth and use of water tub and upright position for birth when compared to disadvantaged women intending to give birth in an OU.

## Methods

### Design

The study was a cohort study with a matched control group. Data were sampled during a 3.5-year period between 2004 and 2008.

### Setting

The study was conducted in the peripheral and relatively sparsely populated North Denmark Region, which provided low-risk women with a free choice of birthplace between two FMUs and two OUs. All four units were publicly financed and cooperated closely on referral and transfer on the basis of multi-disciplinary guidelines.

In Denmark, pregnant women have shared antenatal care provided by a general practitioner and a midwife who are both responsible for screening of pregnant women for risk factors and referral to a higher level of care in case of complications or indications of such. The lines of referral follow regional, multi-diciplinary guidelines. In the North Denmark Region low risk women had the choice of intrapartum care from any of the two FMUs and two OU in the region or a home birth (1%) and they were able to change their decision at any time, including during labour. Proximity/ accessibility has been found to be an important factor for women’s choice of birthplace in the region.

### Freestanding maternity units

The two FMUs were located in the vicinity of two community hospitals, staffed by 4–8 midwives who provided antenatal, intrapartum and postpartum care in a team care model. No on-site obstetrical service was available in the two FMUs, who saw approximately 170 (Hobro FMU) and 130 (Frederikshavn FMU) births a year.

The two units were characterised by one-to-one care and continuous support throughout labour and active encouragement of women to ambulate and use water and music for pain relief and relaxation. Following the Region’s multidisciplinary guidelines for referral and transfer, all FMU women were offered a 20 minute cardiotocography test as a screening for fetal well-being. Midwives and obstetrician agreed on this practice although not fully evidence based as it lowered some medical concerns over perinatal safety and offered increased documentation of fetal well-being at the start of care in labour.

The midwives employed at the FMUs had at least two years of practice experience and multidisciplinary mannequin training in obstetrical emergencies, including ventouse delivery. In case of complications or any indication of them, the women and/or infants were transferred to the nearest OU/ Neonatal Intensive Care Unit (NICU) 25 to 35 minutes away. If possible, FMU midwives accompanied women during transfer and continued care under supervision of an obstetrician in the OU. Please see Table 1 for further information.

**Table 1:** Characteristics of the participation FMUs and OUs

	**The Freestanding Midwifery Units**	**The Obstetric Units**
**Referral to place of birth**	Risk assessment by midwife and general practitioner at all antenatal visits	Risk assessment by midwife and general practitioner at all antenatal visits
	Low risk women self-referred to preferred place of birth (home, FMU, OU). Decision could be changed at any time	Low risk women self-referred to preferred place of birth (home, FMU, OU). Decision could be changed at any time
**Primary intrapartum care provider**	Midwife	Midwife
	In case of transfer, the FMU midwife would accompany the women to an OU and if possible, continued care, supervised by an obstetrician.	In case of complications, the OU midwife would continue care, supervised by an obstetrician.
**Midwifery staff**	Midwives with >2 years of training, working in a team care model. When needed the FMU midwives would assist at the nearest OU if the FMU was not busy.	Midwives with different levels of experience, supervised by consultant midwife. No team care.
	All FMU midwives provided antenatal care one day a week for high and low risk women in the area, regardless of the woman’s choice of birthplace	Most OU midwives provided antenatal care one day a week for high and low risk women in the area
		OU midwives worked in a combination of 8-hour shifts and 24-hour (on-call) shifts.
	The FMU midwives provided intrapartum and out-of-hours post partum care in 24-hour, on-call shifts.	
		No OU midwives provided post partum care
	1–2 FMU midwives provided only antenatal and postnatal care (all women in the area with low risk of post partum complications could be admitted to the postnatal ward).	
**Care concept**	Priority was given to one-to-one care and continuous support in labour. Most women would be cared for by 1(−2) different midwifes during labour.	One-to-one care and continuous support in labour typically not available. Most women would be cared for by 2–3 different midwives during labour
	Active encouragement of ambulation, use of different labour positions and use of water and music for pain relief and relaxation.	Ambulation, use of different labour positions, use of water and music for pain relief and relaxation possible but not routinely encouraged.
	Amniotomy (<5 cm dilatation) and episiotomy could be performed if considered relevant by the midwife	Amniotomy (>5 cm dilatation) and episiotomy could be performed if considered relevant by the midwife as well as oxytocin augmentation of labour (the latter only on basis of local guidelines).
**Cardiotocography (CTG)**	Auscultation.	Auscultation.
	Admission CTG offered to all women. Transfer performed if CTG indicated	No Admission CTG. CTG only used on indication (including epidural analgesia and oxytocin augmentation)
Assistance for emergencies *	The FMUs were hosted by regional hospitals providing 24-hour emergency, on site assistance from anaesthesiologist (day) / capable specialist nurse (evening + night).	Assistance of obstetrician, anaesthesiologist and paediatrician available 24-hour on site / on site during daytime
	All obstetric and paediatric assistance required transfer	
**Transfer**	Ante- and intrapartum referral/transfer to OU on basis of regional, multi-disciplinary guidelines. The FMUs, OUs and ambulance service had well-established routines for ambulance transfer of mother and infant.	OU midwife / consultant midwife/ obstetrician and/or paediatrician would always be contacted by FMU midwife before transfer in order to prepare the admission of the patient.

### Obstetric maternity units

Aalborg University Hospital, located in the regional capital, is a highly specialised hospital offering a specialist OU 24-hour on-site service with approximately 3500 births a year. The unit was staffed by consultant obstetricians, paediatricians, anaesthesiologists and midwives. Vendsyssel Hospital, located in the main town of a municipality of Hjørring, has ten clinical specialities including a generalised paediatric ward and an obstetric unit that provides care for low-risk and most high-risk women (approximately 1400 births a year).

The birthing rooms at both OUs were conventionally equipped with a labour bed as the central feature. Electronic fetal monitoring was not routinely used in births in low risk women. As in the FMUs, birthing pools were available and used both for pain relief and water birth, but one-to-one care and continuous support in labour was typically not available until late in the first stage of labour. Epidural analgesia was available 24 hours a day (used in 10-15% of all births during the study period). In Denmark, midwives are the lead carer for all low risk women including those giving birth in obstetric units.

### Participants

The study included 839 low-risk women intending to give birth in Hobro or Frederikshavn FMUs and a matched control group of 839 low-risk women intending to give birth in the one of the obstetric units at Aalborg University Hospital or Vendsyssel Hospital.

The study included all women admitted in labour to the FMUs on the basis of the regional, multidisciplinary admission criteria during the study period and their individually matched controls, identified among low-risk women intending to give birth in the nearest OU.

### Definition of low risk

Women in the study were categorised as low-risk if they were healthy, had presented in spontaneous labour between 37 + 0 and 41 + 6 weeks of gestation and had no obstetric risk-increasing conditions as outlined in the NICE Intrapartum Care Guidelines [74]. Women with fetal growth retardation in an earlier or in current pregnancy and severe social problems such as substance or drug abuse or a history of child neglect were not eligible for FMU care.

### The matching process

For each participant included in the FMU study group, a data form containing anonymised information on matching data was sent to the project staff at the nearest OU. Control participants were selected from the region’s patient administration system which contains detailed information on all pregnant women in the region. All controls were prospectively identified among the low-risk women admitted to the nearest OU. Matching was performed at the start of care in labour on the following criteria: low-risk status, parity and smoking status, Body Mass Index (BMI), first language, education level, occupation level, and cohabitation status.

### Variables and data measurement

In our overall study of FMU care, Apgar score of <7 at 5 min and caesarean section was defined as primary outcomes to allow for comparison with other studies. An important secondary outcome was spontaneous vaginal birth. These outcomes are reported in [59].

This study compares two models of care for low risk women, both striving to achieve the best perinatal and maternal birth outcome. In this analysis, we focused on the optimum outcome of birth: *a spontaneous, uncomplicated birth leaving both mother and infant in good condition”* as the primary outcome. This outcome was defined as birth following spontaneous onset of labour in 37^th^ to 42^nd^ gestational week leading to spontaneous birth of an infant with a minimum Apgar score of 9 or 10 at 5 minutes and no need of NICU admission. The analysis was based on the intention-to-treat principle and women were excluded if experiencing: shoulder dystocia, third-fourth degree perineal tear, uterine rupture, caesarean section, instrumental delivery, medical augmentation of labour, episiotomy, retained placenta, and bleeding exceeding 500 ml. Participants who during labour had epidural analgesia, CTG monitoring and amniotomy were included if they did not experiencing the mentioned complications or interventions but had a spontaneous vaginal birth with good maternal and perinatal outcome.

Apgar score of <9 at 5 min was chosen as another primary outcome. A 5 min Apgar score of <9 cannot be classified as a poor outcome but in this specific study of freestanding midwifery units, located 50 km (25–35 min of ambulance transfer) away from the nearest obstetric unit, it is an undesirable outcome that would prompt action from the midwife both in term of care for the infant and call for assistance. In some cases immediate transfer to NICU would be needed, in other cases the infant would be kept under increased observation, at least for some hours after birth. Depending on the infant’s 1 and 10 min Apgar Score and general condition, the midwife would have to assess the infant’s risk of e.g. neonatal hypoglycaemia and hypothermia and maybe seek pediatric advice (e.g. via telephone). Furthermore, the routine postnatal care regime for low risk women (discharge few hours after birth or admission to the postnatal ward with only on-call staff between 8 pm and 8 am) may not be considered safe.

Other outcomes related to the infants were: NICU admission <24 h, readmission 0–28 days postpartum, while outcomes related to the mothers were: caesarean section, instrumental delivery, augmentation of labour, intact perineum, third or fourth degree tear, maternal readmission 0–28 days postpartum, epidural analgesia, water birth and upright position for birth.

The intended place of birth at the start of care in labour was considered the exposure.

A number of socio-economic factors have traditionally been used as indicators for women’s social position in society, including length of education, income, occupation, unemployment and level of area deprivation. Our choice of education as explanatory variable was based on both international and Danish findings [3,7,9-11,14,75,76] which have established a clear association between a low level of education and numerous negative health outcomes such a low birth weight and preterm birth.

In Denmark education is free and compulsory from the age of seven to 16 (9 year program). In 2008, 56% of students continued to complete upper-secondary education after 12 or 13 years. Students aiming to continue into a vocational education program (35%) often chose the comprehensive school’s optional 10th year [77]. This provided the basis for dichotomising the women’s school qualifications into “No post-secondary” versus “All types of post-secondary education”. Women with 9–13 years of schooling were categorised into the group “No post-secondary education” if they had not completed or were not undertaking any official training or educational program qualifying for the labour market. Post-secondary education was used as cut-off point, as the absence of labour market qualification increases the risk of unemployment or employment involving manual, physically demanding and/or, unfulfilling labour with low pay.

### Statistical analysis of data

The present study is a secondary analysis, and power calculations are thus not performed. The study strength is however reflected in the confidence intervals.

For the overall study, power calculations and thus sample size was estimated on basis of a number of clinical endpoints in relation to maternal and perinatal morbidity, birth complications and interventions. Due to unexpected closure of the two participating FMU during the data collection period, the study sample size was reduced from the originally planned 1027 women to 839 women in each group. To achieve the highest possible number of women in the FMU group, 289 women that had been admitted to the FMUs between 01.03.2004 and the original study start 01.01.2005 were included in the study and prospectively matched with control participants. This decision was made after thorough revision of the study protocol and only possible because of the highly detailed patient records that were of very good quality. The study implication is discussed later and also in Overgaard et al. 2011[59] (open-access publication of the overall study).

The reduced sample provided power (5% significance level, 80% power) to detect an increase in Apgar score <7 at 5 min from the expected 1.07% in the OU standard care group to 3.1% in the FMU group and a reduction in the incidence rate of caesarean section from 8.8% in the OU group to 5.5% in the FMU group.

The data analysis was carried out by use of STATA statistical software, version 11. Two groups of women with fully comparable obstetric and socio-demographic characteristics were matched on the basis of their intentions regarding birthplace. Women opting for an FMU were matched 1:1 with women who preferred an OU. The analysis was based on an intention-to-treat principle.

For all outcomes a conditional logistic regression grouped on match-pairs was applied to estimate and test the effect of birthplace overall and in education-induced subgroups as well for assessing effect differences between subgroups. For all comparisons, odds ratios with 95% confidence intervals were calculated. All reported P-values were two-sided with a statistical significance level of 5%.

### Data security and ethics

The project was approved by the Danish Data Protection Agency (reference number: 2005-41-5352) and data were treated in strict accordance with Danish legislation on the use of patient data in research [78]. According to this legislation, ethical approval from authorities or participants’ consent is not required for an observational study of this kind.

### Participant characteristics

**Figure 1:**
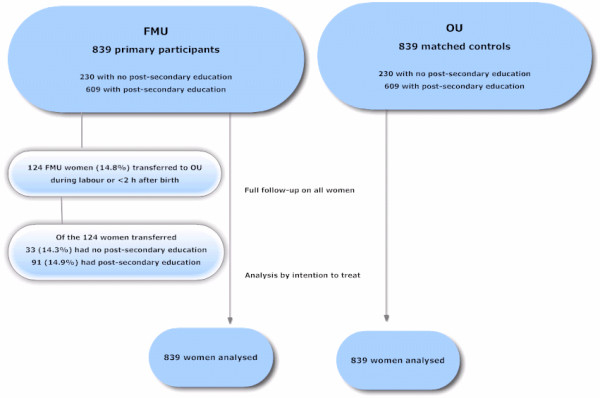
Flow chart.

Each of the two study groups comprised 839 low-risk women, none of whom were lost to follow up (see Figure 1, flow chart).

As shown in Table 2, the matching produced two fully comparable groups in terms of key medical and socio-demographic factors. Almost all women had as their first language a Nordic or West European language (FMU 96%; OU 96.4%) and were married or cohabiting with a partner (FMU 97%; OU 97.4%).

**Table 2:** Participant characteristics

**Characteristics**				
	**FMU**		**OU**	
	**N**	**(%)**	**N**	**(%)**
Low obstetric risk of complications	839	(100)	839	(100)
Primiparas	215	(25.6)	215	(25.6)
Multiparas	624	(74.4)	624	(74.4)
Non-smokers	684	(81.4)	684	(81.4)
Smokers	156	(18.6)	156	(18.6)
First language Nordic or Western European	805	(96.0)	809	(96.4)
Other first language	34	(4.0)	30	(3.6)
No post-secondary education	230	(27.4)	230	(27.4)
Post-secondary education	609	(72.6)	609	(72.6)
Low level of employment*	535	(63.8)	535	(63.8)
High level of employment	304	(36.2)	304	(36.2)
Living with partner	815	(97.1)	819	(97.6)
Living alone	24	(2.9)	20	(2.4)
	**mean**	**(SD)**	**mean**	**(SD)**
BMI	24.2	(3.9)	24.0	(3.9)
Age	29.4	(4.6)	30.2	(4.5)

*Unskilled work, skilled work (vocational), work requiring maximally 2 years of post-secondary training.

Education and income levels in the North Denmark Region were low compared to the Danish population in general [79], conditions which are reflected in the characteristics of the pregnant women in the predominantly rural catchment areas of the two FMUs. Thus, 27.4% of the women had no post-secondary education, 63.8% were had a low level of employment (or were unemployed). Smokers made up 18.6%. Means for BMIs were 24.2 and 24.0, for age 29.4 and 30.2 years in the FMU and OU groups, respectively.

## Results

We analysed the effect of educational level on a range of outcomes, presented in Table 3.

**Table 3:** Outcomes by level of social disadvantage

**Outcome**	**No post-secondary education**		**Post-secondary education**		**Effect ratio**
	230 FMU/ 230 OU OR (95% CI)		609 FMU / 609 OU OR (95% CI)		OR (95% CI)
	N FMU / N OU	FMU / OU	N FMU / N OU	FMU / OU	No post sec./Post sec .
Optimal outcome of birth*	192/156	2.4 (1.5-3.9)	510/434	2.7 (1.9-3.7)	0.9 (0.5-1.6)
Perinatal outcomes					
Apgar score <9/5 min	5/5	1.0 (0.3-3.5)	10/15	0.7 (0.3-1.5)	1.5 (0.3-6.6)
NICU admission >24 hours	7/10	0.7 (0.3-1.8)	11/13	0.8 (0.4-1.9)	0.8 (0.2-3.0)
Infant readmission 0–28 days p.p.	8/7	1.1 (0.4-3.2)	15/28	0.5 (0.3-1.003)	2.1 (0.6-7.0)
Maternal outcomes					
Intact perineum	159/144	1.4 (0.9-2.1)	355/322	1.3 (1.002-1.6)	1.1 (0.7-1.8)
3rd-4th degree perineal tear	5/6	0.8 (0.6-2.7)	14/18	0.8 (0.4-1.6)	1.1 (0.3-4.5)
Maternal readmission 0–28 days p.p.	4/10	0.4 (0.1-1.3)	20/30	0. 7 (0.4-1.2)	0.6 (0.2-2.2)
Interventions					
Caesarean section	6/11	0.5 (0.2-1.5)	13/23	0.5 (0.3-1.1)	1.0 (0.3-3.5)
Instrumental delivery	5/11	0.4 (0.1-1.3)	20/50	0.3 (0.2-0.6)	1.2 (0.3-4.2)
Syntocinon augmentation of labour	19/40	0.4 (0.2-0.7)	50/114	0.3 (0.2-0.5)	1.0 (0.5-2.3)
Pain relief and position for birth:					
Epidural analgesia	10/27	0.3 (0.1-0.7)	25/59	0.3 (0.2-0.6)	0.8 (0.3-2.1)
Water birth	33/15	2.5 (1.3-4.9)	126/56	2.6 (1.8-3.7)	1.0 (0.4-2.0)
Upright position for birth	43/28	1.6 (0.8-3.2)	145/130	1.9 (1.4-2.7)	0.8 (0.4-1.8)

*Defined as uncomplicated birth with spontaneous onset of labour between 37 + 0 and 42 +0 weeks of gestation leading to spontaneous birth of an infant with a minimum Apgar score at 9 or 10 at 5 minutes. No shoulder dystocia, 3–4 degree perineal tear, no bleeding >500 ml, retained placenta, no caesarean section, no instrumental delivery, no medical augmentation of labour, and no episiotomy. (Women having medical analgesia (including epidural analgesia), CTG monitoring and amniotomy are included if not experiencing the mentioned complications or interventions).

### Optimal outcome of birth

Compared to women in the OU group, women in the FMU group were significantly more likely to have an uncomplicated, spontaneous birth with good outcomes for mother and child (OR 2.6; CI 2.0-3.4). This effect was also found for women with post-secondary education: OR 2.7; CI 1.9-3.7 and without post-secondary education: OR 2.4; CI 1.5-3.9 (effect ratio 0.9; CI 0.5-1.6).

### Perinatal outcomes

No significant differences were found between FMU and OU women with respect to Apgar scores <9/5 min, NICU admission >24 hours or infant readmission to hospital 0–28 days postpartum. The same findings applied regarding perinatal outcomes when the two groups of women were compared by level of education. Nor was any significant effect difference between subgroups found.

### Maternal birth outcomes

Women in the FMU group were significantly more likely to have intact perineum (OR 1.3; CI 1.1-1.6) and avoid readmission to hospital during the first four weeks after birth (OR: 0.6; 0.4-0.99).

For intact perineum, similar trends were found for both women with (OR 1.3; CI 1.002-1.6) and without (OR 1.4; CI 0.9-2.1) post-secondary education, but for the latter the result was not significant. Effect ratio was OR 1.0; CI 0.3-3.5. For maternal readmissions, there were similar but non-significant trends for both subgroups of women (effect ratio OR: 0.6; 0.2-2.2).

The occurrence of third or fourth degree tears was similar in both study groups and in both education level subgroups.

### Birth interventions

A caesarean section was significantly less likely in women in the FMU group compared to the OU group (OR 0.5; 95% CI 0.3-0.9). Similar, but non-significant trends were found both for women with (OR 0.5; CI 0.3-1.1) and without (OR 0.5; CI 0.2-1.5) post-secondary education (effect ratio 1.0; CI 0.3-3.5).

Instrumental delivery (OR 0.4; 95% CI 0.2-0.6) and augmentation of labour (OR 0.4; 95% CI 0.3-0.5) were significantly less frequent in women in the FMU group compared to the OU group. There were similar findings by the level of education. In the post-secondary education group: instrumental delivery (OR 0.3; 95% CI 0.2-0.6) and augmentation of labour (OR 0.3; 95% CI 0.2-0.5). Women without post-secondary education: instrumental delivery (OR 0.4; 95% CI 0.1-1.3) and augmentation of labour (OR 0.4; 95% CI 0.2-0.7).

Effect ratio for instrumental delivery was (OR 1.2; CI 0.3-4.2) and for augmentation of labour (OR:1.0; 0.5-2.3).

### Pain relief and position for birth

Overall, epidural analgesia (OR 0.3; CI 0.2-0.5) was significantly less likely among FMU women compared to OU women whereas water birth (OR 2.6; CI 1.9-3.5) and use of an upright position for birth (OR 1.9; CI 1.4-2.5) were significantly more likely.

A significant reduction in use of epidural analgesia was also found both for women with post-secondary education (OR 0.3; CI 0.2-0.6) and for women without post-secondary education (OR 0.3; CI 0.1-0.7), (effect ratio OR 0.8; CI 0.3-2.1).

In the case of water birth a significant increase was seen both for women with post-secondary education (OR 2.6; 1.8-3.7), and women without post-secondary education (OR 2.5; 1.3-4.9), (effect ratio OR 1.0; CI 0.4-2.0).

For the use of an upright position for birth, a significant increase was found in women with post-secondary education (OR 1.9; 1.4-2.7). Similar but insignificant trends were found for women without post-secondary education (OR 1.6; CI 0.8-3.2).

### Other analyses

One hundred and twenty-four women in the FMU group (14.8%) were transferred during the intrapartum period and less than two hours after birth (see figure 1). The rate of transfer was unaffected by the women’s educational status (14.3%; 14.9%).

## Discussion

Freestanding midwifery units form part of the maternal health services in several countries, where they provide women at low risk of obstetric complications with a choice among birthing facilities and more accessible care. In this study, we investigated whether the effect of birthplace on perinatal and maternal morbidity, birth interventions and use of pain relief among low risk women intending birth in two freestanding midwifery units versus two obstetric units in Denmark differed by level of social disadvantage measured by level of education.

### Key results

Overall, women in the FMU group had a higher likelihood of spontaneous, uncomplicated birth with good outcomes for both mother and child compared to women in the matched control group who received the standard OU care. Furthermore, FMU women had a higher likelihood of intact perineum, water birth, and use of an upright position for birth and a lower likelihood of caesarean section, instrumental delivery, augmentation of labour, epidural analgesia for pain relief and maternal hospital readmission. No difference in perinatal outcomes or 3^rd^-4^th^ degree tears was found between groups.

While the level of education is generally found to be high among women opting for out-of-hospital settings for birth [44,58,65-68], we found that as many as 27.4% of the women had no post-secondary education and 63.8% had unskilled work, vocational work or other low level of employment.

For the two subgroups of women with or without post-secondary education, both perinatal and maternal birth outcomes were equal to and more positive for women intending to give birth in an FMU compared to women intending to give birth in an OU.

When we compared women without post-secondary education according to their intended birthplace, the FMU women were found to have a significantly higher likelihood of spontaneous, uncomplicated birth and water birth and a significantly lower likelihood of augmentation of labour and epidural analgesia than the OU women. No differences in perinatal outcomes were detected. Overall the effect of birthplace on birth outcomes did not differ with women’s level of education.

### Study limitations and strengths

This study presents a secondary analysis of the study data. In consideration of the study’s limited power to investigate rare adverse outcomes, we opted for the composite outcome “uncomplicated, spontaneous birth with good outcome for mother and infant”. This outcome defined the optimum outcome of birth and took into account all serious perinatal and maternal morbidity and was inspired by the World Health Organisation’s definition of normal birth [80]. Confidence intervals are provided for ease of interpretation of the study results; they are relatively wide for Apgar scores <9 at 5 min, infant readmission and water birth.

The non-randomised design of the study represents an overall limitation. Although the two study groups were very closely matched and supplementary control for matching factors was performed, the risk of residual confounding and confounding by unknown factors related to women’s choice of birthplace cannot be fully eliminated. The delay of data collection for the FMU participants from 2004 may also entail a risk of bias. The risk was however considered to be minimal as the data collection was individual and project-specific and the study inclusion criteria were very closely observed. The participating unit’s routine statistics were monitored for changes in clinical practices or use of technology and none detected. A subgroup analysis of the 2004-data was performed as part of the overall study to revival potential differences between these data and the main body of data, and reassurance was provided by the finding of concordance of the results. This issue is further discussed in the open-access publication of the overall study results [59].

Our use of education as a proxy for social disadvantage may also be seen as a limitation as no single measurement is likely to be able to capture the full complexity and meaning of a person’s social position and level of social disadvantage. The association between education and birth outcomes is however well documented and believed to be mediated through employment, economical circumstances and psycho-social resources and constraints. In this population, levels of education and income overlapped but we considered education as a key indicator for the following reasons: Danish women have an employment participation rate of 77% which is the highest in EU and among the highest in the world. In a population of pregnant women, education is likely to be a stronger indicator of social position than employment or income because pregnant women are more liable to be (temporarily) outside the active labour force than women overall. Furthermore, education has a stronger influence on women’s ability to obtain, understand and react to knowledge (e.g. when to seek help or ask for advice) and to influence health/pregnancy related behaviours and choices. Income was not considered as useful an outcome as education because most women were employed in the skilled trades or the public sector (extremely few were professionals) and the difference in income would be very small. Women in unskilled jobs would typically have a lower income but some would be able to achieve a higher income than women employed in the public sector. Unemployed women and women receiving social benefit or social pension would have a smaller income but because of the Danish welfare system there financial situation would be better than in many other countries.

The use of project-specific and high-quality data collected at the time of birth is a major strength of the study. The accuracy of key information on women’s educational level and obstetric risk status and on medical outcomes is thus extremely high. Furthermore, no data are missing. Overall, our dataset has unique completeness in comparison to several of the few available controlled studies of FMU care [44,58,81,82] as all eligible women planning to give birth in the FMUs were included and full background data and follow-up on all participants were obtained.

In contrast to some earlier studies of FMU care [81,83], our study setting was advantageous by including four regional units following the same multi-disciplinary practice guidelines with midwives as lead caregivers in all overall setting of a national/public health service. Confounding by difference in caregiver, clinical practice and patient’s ability to pay was thus reduced.

### Interpretation

Our overall findings that perinatal outcomes were comparable for OU and FMU women and that FMU women had fewer interventions corroborate the results of other controlled studies of FMU care[44,81,83-88], only one of which was undertaken in a population of low-income women [81]. Moreover, the results were in line with the results of a large German register study of FMU care [89].

In our restricted sample of healthy low-risk women with spontaneous onset of labour at term after an uncomplicated pregnancy, the positive results of FMU care as compared to OU care were found to hold for both women with post-secondary education and the potentially vulnerable group of FMU women without post-secondary education.

In all cases, FMU women without post-secondary education had comparable and in some respects favourable outcomes when compared to the individually matched group of OU women with the same level of education.

Most importantly, a significantly higher likelihood of “uncomplicated, spontaneous birth with a good outcome for mother and infant” was seen for FMU women with no post-secondary education compared to OU women with no post-secondary education. We found the FMU women were significantly more likely to avoid interventions and epidural analgesia, and to have a water birth and this effect of birthplace did not differ with level of education. Richmond’s contention that water birth is “mainly pursued by educated, middle class women” thus seems unfounded in this context[90]. Neither did effect ratio differences indicate that option of having a water birth, epidural analgesia or using upright positions for birth as suggested by other studies were less open to disadvantaged women [19-21,90].

In contrast to studies of out-of-OU birth in general, university- or college-educated women constituted only a minority in this study, while women with no post-secondary education or vocational training comprised the majority. Overall, the level of education among women who chose FMU care was considerably lower than in most studies, a difference that may be ascribed to two factors: the location of the FMUs in peripheral and partly rural areas where the level of education is among the lowest in Denmark and, secondly, to the FMUs’ offer of care close to home. For our sample of low risk women, the results provide no support for the claim that women pursue different birth models and that their aims and wants for pregnancy and birth vary according to their socio-demographic backgrounds [33,34]. Neither was such a claim supported by the responses to our questionnaire survey exploring the birth experiences and care perceptions of the participating women [63]. As the Danish Birth Register does not include data on women’s education [91], we were unable to establish whether the choice of local FMU care varied with the women’s level of education. An investigation of potential inequalities in relation to women’s choice of birthplace, including their knowledge of options available to them, would be relevant.

Considering that Denmark has seen a rising trend for markers of social inequality in birth outcomes such as low birth weight and infant mortality [76,92], we consider it an important finding that birth outcomes of FMU care for low risk women at term did not differ by women’s level of education.

The results indicate that the strict low-risk criteria used for this study (reflecting the NICE guidelines for intrapartum care [74]) are helpful in selecting a group of women with low risk of obstetric complications for whom FMU care is very suitable.

The questionnaire survey of the participating women’s birth experience and care perceptions documented significantly improved outcomes in the FMU group compared to the OU group and found a mitigating effect of FMU care on the effects of social disadvantage on birth experience [63]. This ability of the FMUs to serve disadvantaged women particularly well was not seen in this study of clinical birth outcomes, where, as compared to OUs, the advantaged and disadvantaged women were found to benefit equally well from FMU care.

In their qualitative study of inequality in maternity care services Hart & Lockley found an absence of clear and specific strategies to combat inequality in maternity care and a pervading assumption that the concept of woman-centred care would provide an appropriate and focused response to the problem of social inequality[40]. At the time of data collection for this study, social inequality in birth outcomes received limited attention in both national [93] and regional [94] recommendations for maternity care. The initiatives outlines were directed towards women with severe problems such as drug addiction while the concept of individualised and patient-centred care was much stronger emphasised. It thus seems unlikely that the absence of signs of overall social inequality in perinatal and maternal outcomes found in this study should be credited to a special focus among Danish midwives on inequality of care or special strategies or initiatives directed towards social inequality in the maternity care sector.

However, as documented by Cliff, Danish midwives’ have a longstanding tradition of caring for women from all social groups and focusing on the impact of social disadvantage for women and infants[95,96]. Although our earlier study [63] found social disadvantage to be a factor in women’s birth experience and perception of care in OUs compared to FMUs, maternity units in the North Denmark Region that were not struggling with understaffing or shortage of midwives may very well be capable of providing clinical care that were sensitive to the impact of social disadvantage on health of women’s and infants. Studies of social inequalities in care provision are few, but little socioeconomic variation has also been found for neonatal care [97].

Overall, we found that FMUs were capable of offering clear benefits for disadvantaged, low-risk women with no obvious drawbacks while the group of more advantaged women was also well catered for. In a public health perspective, FMU care holds great potential for improvement of birth outcomes for the population of low-risk women. It is the responsibility of policy makers and health professionals to consider how FMU care can be made accessible to more low-risk women, and how women of all social positions can be supported in making an informed choice of birthplace.

### Generalisability

It has been convincingly argued that a country’s level of social inequality is reflected in the health of its population [98]. Any generalisation of the study results should take into account that the Danish levels of social equality are among the highest in the world while rates of perinatal and maternal mortality and morbidity are among the lowest [1,99]. The socially disadvantaged women in this study may therefore have been less burdened than women of comparable social status in countries with greater social inequality and/or less comprehensive welfare systems. Furthermore, the free access to maternity care services may have mitigated the effect of social inequality in birth outcomes. Although a straightforward association between the Nordic welfare model and a low degree of social inequality in health has not been demonstrated, these factors should be taken into consideration when generalising the results of the study.

With regard to the ability of FMUs to serve disadvantaged women, it should be noted that the FMU care concept was based on strict, multi-disciplinary criteria for referral and transfer of women, indicating growth retardation, substance or drug abuse, and social factors such as a history of child neglect as risk factors. Neither smoking nor social factors such as poor housing, dependence on social benefit or social pension, dyslexia, or young age were however considered as risk factors on their own.

## Conclusions

The present study of FMU versus OU care with midwives as lead caregivers in a public health care system identified several benefits of FMU care for the mother with no additional risk to the infant.

Women intending to give birth in an FMU were found to have significantly increased likelihood of uncomplicated, spontaneous birth with good outcomes for mother and infant. The positive effect of FMU care on perinatal and maternal morbidity, birth interventions and use of pain relief was not found to differ by women’s level of social disadvantage.

The strict risk assessment criteria used in the study proved useful in defining a group of women with low risk of obstetric complications. As results for both perinatal and maternal outcomes for women with no post-secondary education intending to give birth in an FMU were similar to or favourable in comparison to the results for women with no post-secondary education intending to give birth in an OU, FMU care must be considered as appropriate for this group of women as for other women with low risk of obstetric complications.

The potential of FMU care to improve maternal health without increasing perinatal risk lead us to suggest that the option of FMU care is made available to low-risk women in all social groups and that all women are provided adequate information about different care models and their benefits and harms in order that they are enabled to make an informed decision about where they want to give birth.

## Competing interests

The authors declare that they have no competing interests.

## Authors' contributions

CO is responsible for the study’s conceptual design, designed the data collection tools, monitored all data collection, and cleaned the data. She also participated in the data analysis and the interpretation of data, drafted and revised the article after comments and wrote the final version. MFG participated in the data analysis and revised the article for important intellectual content. JS participated in the interpretation of data and revised the manuscript for important intellectual content. All of the authors read and approved the final version that was submitted for publication.

Findings of a matched cohort study of two different models of intrapartum care for low risk women.

## Pre-publication history

The pre-publication history for this paper can be accessed here:

http://www.biomedcentral.com/1471-2458/12/478/prepub
